# Drug delivery strategies to improve the treatment of corneal disorders

**DOI:** 10.1016/j.heliyon.2025.e41881

**Published:** 2025-01-10

**Authors:** Mahsa Fallah Tafti, Zeinab Fayyaz, Hossein Aghamollaei, Khosrow Jadidi, Shahab Faghihi

**Affiliations:** a Stem Cell and Regenerative Medicine Group, National Institute of Genetic Engineering and Biotechnology, Tehran 14965/161, Iran; b Edison Biotechnology Institute, Ohio University, Athens, OH, 45701, USA; c Chemical Injuries Research Center, Systems Biology and Poisonings Institute, Baqiyatallah University of Medical Sciences, Tehran, Iran; d Vision Health Research Center, Semnan University of Medical Sciences, Semnan, Iran

**Keywords:** Corneal disorders, Drug delivery systems, Personalized medicine, 3D printing

## Abstract

Anterior eye disorders including dry eye syndrome, keratitis, chemical burns, and trauma have varying prevalence rates in the world. Classical dosage forms based-topical ophthalmic drugs are popular treatments for managing corneal diseases. However, current dosage forms of ocular drugs can be associated with major challenges such as the short retention time in the presence of ocular barriers. Developing alternative therapeutic methods is required to overcome drug bioavailability from ocular barriers. Nanocarriers are major platforms and promising candidates for the administration of ophthalmic drugs in an adjustable manner. This paper briefly introduces the advantages, disadvantages, and characteristics of delivery systems for the treatment of corneal diseases. Additionally, advanced technologies such as 3D printing are being considered to fabricate ocular drug carriers and determine drug dosages for personalized treatment. This comprehensive review is gathered through multiple databases such as Google Scholar, PubMed, and Web of Science. It explores information around "ocular drug delivery systems'', "nano-based drug delivery systems'', "engineered nanocarriers'', and "advanced technologies to fabricate personalized drug delivery systems''.

## Highlights

• The delivery systems for the treatment of corneal diseases are introduced.
• Nanocarriers are promising platforms for customizable ophthalmic medication and treatment.
• 3D and 4D printing can be used to design delivery platforms based on corneal damage.
• Personalized platforms offer an attractive route to study and improve therapeutic precision and effectiveness.

## Graphical abstract

**Figure:**
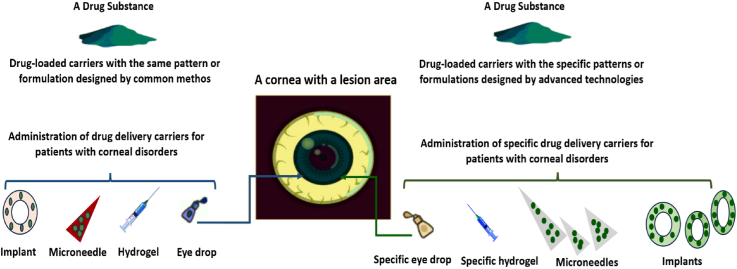

## 1 Introduction

The normal cornea is a connective tissue with dome-shaped, transparent avascular, and compact multilayer structure in the eye's front portion [1]. This tissue consists of five layers including epithelium, Bowman's layer, stroma, Descemet's membrane, and endothelium [[1], [2], [3]]. The cornea serves important functions such as protecting the eye, contributing to the eye's refractive power, focusing light rays on the retina with minimum scatter, and preventing optical degradation [4]. Infectious keratitis, corneal perforation, melting, corneal scarring, vascularization, haze, and corneal dystrophies can be significant causes of visual impairments resulting in reduced corneal transparency, vision loss, and blindness. However, there is also a variation in the prevalence and epidemiology of corneal diseases according to region [[5], [6], [7]]. For example, the environmental risk factors such as UV light exportation during corneal collagen cross-linking (CXL), contact lens wear, and refractive surgeries (e.g., LASIK (laser-assisted in situ keratomileusis, small incision lenticule extraction (SMILE)) or Photoreactive keratectomy (PRK) can contribute to the development of corneal disorders [[8], [9], [10], [11]]. For example, spontaneous persistent epithelial defects (PEDs) can appear following surgical approaches such as PRK. After surgery approaches, transforming growth factor beta isoforms (TGFβ-1, TGFβ-2, or TGFβ-3) can localize in the corneal stroma at PEDs and promote myofibroblasts development from precursor cells [[12], [13], [14]]. The above findings favor previous pre-clinical studies conducted on rabbit models with PRK [15,16]. According to the experimental observations, spontaneous PED signs with the presence of myofibroblasts were indicated 7 days post-surgery in the superficial stroma at PED. Some myofibroblast marker alpha-smooth muscle actin (α-SMA) with TGFβ-3 localized to the stroma at PEDs. After PRK, all three TGFβ isoforms could be detected in the stroma at PEDs. This pathological mechanism likely prompts corneal scarring, opacity, and visual impairment.

Current therapeutic methods include non-invasive (e.g. topical eye drops, ocular emulsion, suspension, ointment, and polymeric gels). However, these approaches may be associated with limitations due to intricate anatomical and physiological barriers [[17], [18], [19], [20]]. There are physical methods for improving ocular bioavailability, such as formulations in suspensions (Zigran(®)), solutions (Zymaxid(™)), and gels (Akten®), as well as chemical methods such as prodrugs (Xalatan™), and soft drugs [21]. Soft drugs are referred as chemical compounds like anti-inflammatory corticosteroids that can be administrated locally at the desired eye sites [22].

Nano-based systems have been investigated for disease treatment [23]. Various delivery systems have been applied precisely to encapsulate, transport, and deliver chemical drugs to reduce complications. Based on the type of ocular diseases, delivery systems can be administrated as eye drops, injections, etc [[24], [25], [26]]. Several kinds of delivery systems for ophthalmic diseases are available, including nanoparticles, liposomes, dendrimers, niosomes, hydrogels, contact lenses, punctal plugs, and microneedles [17,27]. These carrier systems have the potential to penetrate the corneal membrane and deliver cargos to the defective parts of the cornea [28]. Therefore, to minimize side effects, it is important to improve drug efficiency and reduce the amount of precorneal drugs [29].

Moreover, drug delivery systems based on nanocarriers or ocular devices are considered for managing ocular diseases. Notably, developing advanced and customized ocular delivery systems would also be beneficial. This review examines the applications of nanocarriers and ocular devices for treating anterior segment diseases of the eye, particularly the cornea. Previous studies-based delivery systems for managing anterior ocular disorders are also evaluated. Finally, advanced technologies, such as 3D printing, may be used to administer drug dosages more precisely, as well as fabricate delivery systems based on an individual's damaged corneal properties [[30], [31], [32]].

## 2 Managing corneal disorders with topical drugs

The eye's surface can be affected by corneal diseases or environmental factors, potentially impairing vision or leading to blindness in some cases [33,34]. Current topical ocular drug-based non-invasive methods include eye drops, such as non-steroidal anti-inflammatory drugs (NSAIDs), steroidal (SAID) drugs, and autologous serum. These drugs are effective in healing ocular surface disorders such as burnt corneas, infectious inflammations, and neovascularization [35,36].

In (Table 1) some examples of current topical drugs for treating other corneal disorders and diseases are mentioned.

**Table 1:** Some of the current therapeutic methods for treating corneal disorders and diseases.

Corneal diseases	Corneal impairments	Current non-invasive medications	Ref.
Corneal trauma	Ocular trauma is caused by an injury due to corneal abrasions and foreign bodies commonly. The severity and symptoms of ocular trauma disorders can include pain, corneal perforation, corneal laceration, recurrent erosion, corneal opacity, and even pose a threat to vision. Bacterial keratitis, corneal ulcer, and PED may be the other complications of corneal injuries.	- To alleviate inflammation and corneal wound healing, topical NSAIDs such as diclofenac eye drops may be effective.	[37,38]
Microbial Keratitis	It is a leading cause of localized pain, corneal scarring, neovascularization and can ultimately result in blindness due to various infectious factors. For example, NK is a rare degenerative disease that can be caused by HSV or herpes zoster infection. It is diagnosed by a reduction or absence of corneal sensitivity. The symptoms are dysfunction of corneal healing process, stromal ulcers, corneal epithelium disruption, impaired healing, corneal opacity and, the risk of melting and perforation. These symptoms can result in an irreversible deficit of vision.	- Antiviral therapy containing systemic drugs as well as systemic and topical drugs such as trifluridine and ganciclovir gel are demonstrated.	[39]
Ocular chemical burns	Chemical burns vary based on the range of penetration of the chemical substances in the front surface of the eye and the severity of their symptoms. Causing LSCD may occur as a result of an alkali burn. This disorder may occur opacification and neovascularization of the cornea.	- Biological fluids such as autologous serum, umbilical cord blood serum, PRP, and amniotic membrane suspension with a rich source of growth factors and angiogenic anti‐fibrotic effects to promote wound healing may be effective in diminishing opacity and corneal neovascularization. - Corticosteroids such as triamcinolone can prescription too	[[40], [41], [42], [43], [44], [45], [46], [47], [48]]
DED or KCS	DES or KCS is a chronic and a multifactorial disease caused by insufficient production of tears in the eye. Common ocular symptoms of DES include eye redness, ocular discomfort, blurred vision and instability of the tear membrane which causes to the damaged ocular surface. Inflammation and oxidative stress play crucial role in the pathogenesis of this disease. Prolonged wear of contact lenses and exposure to weather conditions with low quality and humidity are risk factors for this disease. Additionally, DED can be caused by medical problems such as lupus, scleroderma, and Sjogren's syndrome.	- CsA is known as an FDA-approved drug with immunosuppressive and anti-inflammatory properties. - Topical corticosteroids to reduce inflammation in a short time are also used. - Autologous serum tears have similar properties of biochemical and mechanical with normal aqueous tears which can improve symptoms and signs of severe or refractory in patients.	[[49], [50], [51], [52], [53], [54], [55]]

**Abbreviations:** PED: persistent corneal epithelial defects, NSAIDs: non-steroidal anti-inflammatory drugs, NK: Neurotrophic keratitis, HSV: herpes simplex virus, LSCD: limbal stem cell deficiency, PRP: Platelet-rich plasma, DED: dry eye disease, KCS: keratoconjunctivitis sicca, CsA**:** Cyclosporin A.

Some topical drugs are listed in Table 1. However, topical drugs may present challenges, including corneal epithelial injuries, ulceration, and perforation [37]. Moreover, it is important to note that biological mechanisms, such as eyelid movement, tear turnover, and drainage can significantly affect the rapid elimination of topical drugs from the eye surface [17,35,[56], [57], [58]]. Furthermore, topical drug administration may be associated with low bioavailability, poor solubility, limited shelf life (insufficient in vitro stability), short half-life (insufficient in vivo stability), lack of large-scale production, and necessity for targeted delivery due to ocular barriers [59,60]. In commercial formulations, mucoadhesive properties and contact duration with corneal and conjunctival tissues are diminished. The steroid responses of these agents can increase the risk of cataracts and intraocular pressure [42,43,[61], [62], [63]]. Lack of selectivity, short retention time, and low corneal permeability are the other challenges of topical drugs [20,64,65]. Additional side effects may include the presence of cytotoxic or antigenic/immunogenic agents in patients [65]. Thus, current topical approaches may not be appropriate for the long-term treatment of moderate to severe cases [42,51,62].

## 3 Ocular barriers

**Fig. 1:**
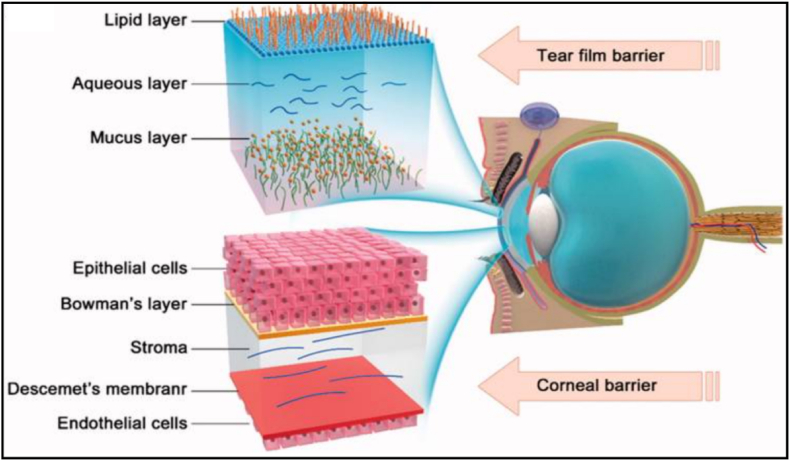
Key bio-barriers of the anterior segment of the eye (Reprinted with some modifications from Ref. [70]).

The eye stands as a well-preserved structure that effectively hinders the penetration of drugs to their intended targets by biological barriers including precorneal, corneal, and blood-aqueous barrier [57]. The cornea, a highly specialized multilayer tissue with a thickness of approximately 0.5 mm, is characterized by its transparency and avascular nature. Three main corneal layers including epithelium-stroma-endothelium are formed equivalent to a fat-water-fat structure. Matured corneal epithelium, serving as a tight diffusion barrier for drug delivery, is equipped with paracellular pores measuring 2.0 nm in diameter. The lipophilic structure of the epithelium layer is a barrier for 90 % of hydrophilic drugs. Conversely, this layer acts as an obstacle for 10 % of hydrophobic drugs [57,58,[66], [67], [68]]. Similarly, the corneal endothelium layer, comprising a monolayer of the cells, can serve as a hindrance to the entry of hydrophilic drug molecules. However, the transfer of hydrophilic drug molecules into these layers is restricted by the presence of the epithelial and endothelial layers. On the other hand, diffusion of hydrophobic drug molecules is inhibited by the corneal stroma regarding their hydrophilic structure. The blood-aqueous barrier is formed by uveal capillary endothelium and ciliary epithelium, effectively restricting the passage of medications from the systemic circulation to the anterior chamber [69]. The structure of important ocular surface bio-barriers including tear film and cornea is shown in (Fig. 1) [70].

## 4 Ocular devices

Ophthalmic devices for ocular disorders have been employed. For instance, punctal plugs are miniature non-invasive medical implants, ranging in size from 2 to 5 mm that prevent the drainage of tears placed in the tear ducts for patients with dry eye syndrome [71]. Punctual plugs as a safe and effective treatment to deliver the drug in a sustained manner are also suggested [72,73].

Using mild electric charges, iontophoresis is a non-invasive method that effectively enhances delivery of drugs through biological barriers. They can be used for drug permeation for anterior and posterior ocular disorders. For example, trans-corneal iontophoresis can be utilized to treat anterior segment disorders, including corneal ulcers, dry eye disease, ocular inflammation, keratitis, and ocular uveitis. However, there may be potential complications associated with this technique, including discomfort and a burning sensation [71,74].

Contact lenses are discs with curved shapes that cover the cornea. Based on material components they have various types, including hard lenses, soft lenses, and silicon lenses. In general, increasing the bioavailability of these lenses is suitable for controlling drug release. Prolonged use of contact lenses can lead to corneal toxicity [[75], [76], [77], [78], [79], [80]]. Prosthetic Replacement of the Ocular Surface Ecosystem (PROSE) lens is an effective drug delivery device. This lens is efficient in improving vision in cases of corneal irregularities and chronic ocular surface diseases associated with corneal neovascularization, Stevens-Johnson syndrome/toxic epidermal necrolysis [[81], [82], [83]].

Collagen corneal shields are similar to ocular lenses and are composed of collagen sourced from bovine or porcine origins [84]. Drugs can be placed into collagen shields, which dissolve and form a lubricating layer to promote epithelial repair and progressive drug release [85]. Drugs can reversibly attach to these reservoirs for regulated release, lowering the risk of systemic toxicity [84]. However, because of their opacity, they may result in impaired vision [86,87]. A straightforward application approach for treating corneal problems such as neovascularization, injuries, dry eye, and infections is provided by nanowafers, which are tiny circular discs or membranes [[88], [89], [90]]. Nanowafers are transparent and can be applied to the eye with the fingertip, withstand continuous blinking. Gradual drug release from the nanowafers prolongs drug retention on the ocular surface and enhances drug absorption [91].

To reduce the side effects of topical cysteamine (Cys) eye drops, such as redness, ocular pain, and inflammation due to frequent administration, a clinically translatable Cys nanowafer (Cys-NW) was fabricated [92]. In vivo experiments in cystinosin knockout mice revealed that Cys-NW with 10 μg Cys, administered once daily, had more therapeutic efficacy than 44 μg Cys delivered as topical eye drops twice daily. Moreover, Cys-NW maintained Cys stability for up to 4 months at room temperature, whereas topical Cys eye drops require freezing or refrigeration and remain effective for only one week. Due to its safety profile, prolonged drug stability, and superior therapeutic efficacy at room temperature, Cys-NW for human application in clinical trials can be suggested.

Through minimally invasive patches, microneedles can be useful for localized drug delivery. Microneedles can deliver both hydrophilic and hydrophobic drugs in precision injections by overcoming the ocular barriers [[93], [94], [95], [96], [97]].

**Fig. 2:**
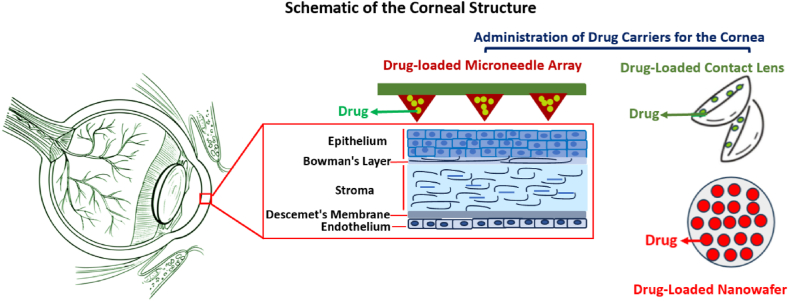
Administration of drug delivery carriers to be used as the corneal segments and provide suitable therapeutic approaches for corneal disorders. (www.vecteezy.com).

Fig. 2, presents a schematic of drug delivery carriers such as contact lenses, nanowafers, and microneedles and their application in corneal disorder treatment.

Some of the ophthalmic drug delivery systems for managing ocular disorders are depicted in (Table 2).

**Table 2:** A summary of evaluated ophthalmic systems for managing ocular surface disorders.

Ocular systems	Drug	Role of drug	Administration	Purpose of Study	Pre-clinical/clinical studies	Advantages	Potential issues/limitations	Ref.
Drug-depository contact lens (DDCL)	Moxifloxacin	An antimicrobial drug	Insertion	-Increasing the time of drug interaction with corneal damaged site - Evaluating the therapeutic efficiency of DDCLs combined with an antimicrobial drug to reduce bacterial keratitis in patients.	Patients with bacterial keratitis In two groups (18 cases with topical antimicrobial drug & 17 cases with DDLC-antimicrobial drug)	- Greater reduction in pain score in patients treated with DDCL-antimicrobial drug compared to group with topical drug treatment during the follow-up period (*p* < 0.05) - Faster disease healing with DDLC-drug due to more drug availability in the lesion site of the cornea compared to topical drug. - Possible reduction in drug consumption by patients treated with DDLC.	More investigations needed for ulcer patients. - Evaluation of drug availability in damaged corneal sites for other keratitis (e,g. protozoal and mycotic).	[98]
Collagen punctal plug			Insertion	Administration of a temporal collagen punctal plug to improve dry eye disease and reduce intraocular pressure	Human patients with dry eye disease and primary open-angle glaucoma (Case group: 33 patients that received glaucoma drugs in the presence of a temporary collagen plug) and (control group with 33 patients that used glaucoma drugs received without punctal plug)	- Improvement in dry eye disease in the case group in comparison with the control group (*p* < 0.001). - Significant reduction of intraocular pressure in the case group compared to the control group.	Short time follow-up - The counterpart eye of each patient should have been considered as control group.	[99]
Collagen shields	Voriconazole	A drug to treatment of fungal keratitis	Insertion	An optimized drug delivery system production	Rabbit eyes	- Higher antifungal efficacy and trans-corneal permeation with the optimized formula than drug solution.	N/A	[100]
Double-layered microneedles	DC101/diclofenac	An anti-angiogenic monoclonal antibody/anti-inflammatory compound	Self-implantable	Evaluating the efficiency and safety of an eye patch fabricated of micro-drug-reservoirs to control drug release into the ocular surface tissue.	Mouse eyes	- Efficient non-invasive home-based treatment for various eye diseases. - Improved corneal neovascularization healing in comparison with topical eye drops. -Providing therapeutic efficacy by fast and sustained release of medications.	N/A	[101]
Polyvinyl alcohol (PVA)- Polyvinyl pyrrolidone (PVP)- Microneedles	Besifloxacin	An antimicrobial drug	Insertion	Evaluation of the polymeric microneedles to deliver besifloxacin	In vitro/Ex vivo infection models	- Higher permeation and deposition of besifloxacin-loaded microneedles (for 5 min) in the cornea compared to free besifloxacin solution (*p* < 0.05). - Superior antibacterial activity in infected corneas with *Staphylococcus aureus* than free besifloxacin.	The reduction of besifloxacin dosage through microneedles should be investigated.	[102]

## 5 The significance of nanotechnology-based drug delivery systems for ocular applications

Traditional ocular dosage forms, such as eye drops, may not be well-suited for treating ocular disorders [55]. Moreover, administrating drugs through the conventional dosage might lead to inactivation or have adverse effects on non-targeted tissues [60,103]. In general, drug delivery systems have shown remarkable efficacy in the treatment of eye diseases [[104], [105], [106]]. Notably, nanotechnology-based drug delivery systems offer alternative strategies to improve drug permeation, optimize and control drug delivery to specific ocular tissue sites, and prolong drug release [107,108].

Substances such as drugs can be loaded into nanocarriers via physical encapsulation, adsorption, or covalent attachment [60,109,110]. Nanocarriers have shown significant potential in the treatment of glaucoma, dry eye, and fungal keratitis [111]. In general, nano-based delivery systems improve therapeutic properties, increase drug solubility, and absorption. For instance, the utilization of drug-loaded carriers to enhance solubility and protect against rapid degradation has been proven. Furthermore, these systems diminish required dosages by enhancing the concentration of drugs in target tissues [[112], [113], [114]]. Essential attributes of effective drug delivery systems consist of favorable biocompatibility, considerable biodegradability, minimal cytotoxicity, and non-immunogenicity. Besides, features like controlled drug release, lysosome escape, swift endocytosis, and high drug-carrying capacity are the other advantageous aspects of nano-based drug delivery systems [[115], [116], [117]]. Regarding the intended design of nanocarriers, it is important to consider their physicochemical attributes, such as particle size, particle shape, surface properties, and chemical composition [60]. A nanoparticle within the range of 5–200 nm can easily evade renal clearance and the reticuloendothelial system during blood circulation. Thus, this size range contributes to their longer retention time in the bloodstream and higher blood concentration [118]. Factors like biological activity, biodistribution, speed of internalization into target cells, and clearance may be influenced by nanomaterial shapes (e.g., spheres, rods, tubes, fibers, and disks). In this case, comparing rod nanoparticles and those that are like fiber has indicated that endocytosis of the spherical shape of nanoparticles is easier and quicker [119]. Furthermore, the surface characteristics of nanomaterials, encompassing surface area, pores, and charge can impact processes like phagocytosis, blood circulation, and biodistribution [120]. In the case of these systems, electrostatic interactions between the positively charged surface and the negatively charged surface of the corneal epithelium prolong drug retention [57].

### 5.1 Solid-lipid-nanoparticles (SLNs)

SLNs are colloidal delivery systems consisting of a solid lipid matrix with a perfect crystal lattice structure, ranging in size from 10 to 500 nm. These nanosystems can encapsulate both hydrophilic and hydrophobic drugs, enabling them to effectively traverse biological barriers. Notable properties of SLNs include controlled drug release, targeted drug delivery, long-term stability, and biocompatibility. Moreover, their large-scale production is feasible. Due to their lipophilic nature, SLNs can cross the epithelial barrier, blood-aqueous barrier, and blood-retinal barriers of the eye [[121], [122], [123]].

Patel et al. [63] developed SLNs containing besifloxacin hydrochloride (BSF@SLN) and optimized to improve antibiotic absorption via passive diffusion in the eye. Compared to Besix eye drops (a commercialized drug), the improved ophthalmic nanoformulation proved to be 2.76 times more effective against bacteria. BSF@SLN ophthalmic nanoformulations were also compared to Besix in an in vivo eye irritation study and demonstrated substantial pharmacological effects.

### 5.2 Nanostructured lipid carriers (NLCs)

NLCs are the second generation of the SLN with non-immunogenic, compatible, and biocompatible properties, ranging in size from 50 to 1000 nm. NLCs are formed from a mix of liquid lipids with solid lipids, preventing crystallization of lipids. Their drug-loading capacity is higher and more efficient in comparison with the SLNs. The existence of liquid lipids in NLCs may be the underlying reason for this feature [58,[124], [125], [126]].

Kumari et al. [127] evaluated an eye drop formulation of dexamethasone-loaded NLC for ophthalmic applications during in vitro and ex vivo studies. Based on observations from superior tolerability and internalization capability in human corneal epithelial cells and ex vivo porcine cornea experiments, this ophthalmic formulation may be applicable to the eye surface distribution. Besides, the biomarkers of inflammation in dexamethasone-loaded NLC were assessed using ELISA. As showed a five-fold decrease in TNF-α production compared to the free dexamethasone solution. To sum up, NLC formulation may be a viable alternative for the topical management of dry eye disease due to its simplicity, scalability, and efficacy.

### 5.3 Niosomes

Niosomes are composed of non-ionic surfactant vesicles. They are suitable for delivering both hydrophilic and lipophilic drugs. The significant properties of these vesicles are biodegradability, biocompatibility, and non-immunogenicity. In addition, they can enhance corneal permeability while reducing drug clearance in the aqueous humor. However, it is important to note that they have some limitations, such as aggregation, fusion, limited shelf life, and potential eye irritation [[128], [129], [130]].

In a prior study by Zeng et al. [128] Tacrolimus (FK506) was administered to patients unresponsive to steroids and cyclosporine to prevent the rejection of corneal allograft. The hydrophobicity, molecular weight, and physiological and anatomical limitations hampered FK506's ocular distribution. By combining niosomes with mucoadhesive hyaluronic acid (HA), FK506HA-coated niosomes were developed. FK506HA-coated niosomes were compared to HA solution and non-coated niosomes using surface plasmon resonance to investigate the mucoadhesion. In rabbits, HA-coated niosomes significantly extended the residence time of FK506 in the precorneal area compared to suspensions or non-coated niosomes. Pharmacokinetic studies of aqueous humor revealed a greater area under the curve for HA-coated niosomes than for suspensions and non-coated niosomes. Using a confocal laser scanning microscope, the hybrid delivery system for FK506 demonstrated synergistic improvement in corneal permeability. By improving mucoadhesion, aqueous humor pharmacokinetics, precorneal retention, and transcorneal permeability, the hybrid approach was shown to enhance FK506 ocular delivery. Collectively, HA-coated niosomes may offer a potential method for FK506 administration in ocular applications.

### 5.4 Liposomes

Liposomes are in the form of either nanovesicular or microvesicular structures of different sizes. Their structure comprises one or more phospholipid bilayers that enclose an aqueous core. Due to their chemical structures, liposomes are capable of encapsulating both hydrophobic and hydrophilic drugs. They have excellent biocompatibility and a cell membrane-like structure. However, concerning the mononuclear phagocytic system or reticuloendothelial system as clearance mechanisms, they have some limitations, such as limited capacity for drug loading, sterilization issues, and short half-life [121].

Liposomes have considerable potential for administering ophthalmic medications since they are small, biocompatible, and capable of delivering drugs with multiple polarities simultaneously. The physicochemical characteristics of a liposome formulation encapsulating Bevacizumab and Dexamethasone were investigated for ocular delivery topically [131]. In the preparation of liposomes, thin-film hydration and extrusion with an optimized formulation were used. The formulation was assessed in wound healing, and sustained release of Bevacizumab from the liposomes was observed. A topical eye drops drug delivery system capable of co-loading Dexamethasone and Bevacizumab was developed and evaluated for delivering ocular medications.

### 5.5 Cubosomes

Cubosomes comprise amphiphilic structures of the carbon chain and are typically less than 500 nm in size. They have a lipid bilayer with a high surface area, which can enhance their efficiency by improving the residence time and bioavailability of hydrophilic and hydrophobic ocular medications with different solubilities [[132], [133], [134], [135], [136]].

In a previous investigation [137], cubosomes containing the antibiotic moxifloxacin hydrochloride were produced using glycerol monooleate and poloxamer 407 to treat conjunctivitis. To prepare moxifloxacin cubogels, optimized cubosomes were dispersed in a cold in-situ gelling system. Compared to a traditional formulation, moxifloxacin cubogel absorbed 81 % through the goat cornea in an in vitro corneal permeation study. Antibacterial and histological studies demonstrated the safety of the formulations selected for ocular application. Based on its enhanced permeability and prolonged release properties, moxifloxacin hydrochloride cubogel can be suggested to serve as a viable alternative to traditional eye drops.

### 5.6 Electrospun nanofibers

Electrospun nanofibers, as an alternative delivery system, offer the advantage of adapting to the eye surface while facilitating sustained release [138,139]. Sangole et al. [140] produced a polymeric ocular insert of levofloxacin using electrospinning technology. Electrospun nanofiber sheets were fabricated using hydroxypropyl cellulose and polycaprolactone polymer. In vitro examination of drug release demonstrated an 8-h release of the medication, with 99 % efficiency. Nanocomposite sheets were formulated to reduce dosing frequency and improve patient compliance through the prolonged release of levofloxacin in ocular diseases.

### 5.7 In-situ gelling systems

These systems involve smart hydrogels that respond to environmental stimuli such as temperature, pH, ions, and ultrasound waves. This responsiveness triggers a sol-gel phase transition, resulting in the formation of a viscoelastic gel. Notably, these systems offer advantages including sustained drug release, prolonged drug contact with the cornea, and enhanced bioavailability compared to conventional eye drops. However, it's worth mentioning that these systems are associated with limitations such as potential blurred vision and the possibility of eyelid adhesion [36,80,141].

Chitosan-carbomer nanoparticles were loaded with timolol and incorporated into a carbomer-based pH-responsive in-situ gel system using the polyelectrolyte complexation method previously [142]. Nanoparticle-laden in-situ gels were analyzed under physiological conditions. Briefly, in-situ gels containing nanoparticles exhibited more mucoadhesive characteristics. In-situ gels containing nanoparticles significantly reduced intraocular pressure compared with timolol solution and nanoparticles alone. Furthermore, the in-situ gelling system significantly reduced intraocular pressure over a longer duration than nanoparticles and timolol solution. Accordingly, the formulated in-situ nanoparticle gel was an effective carrier for ocular drugs. In-situ nanoparticle gelling systems may enhance therapeutic effectiveness by slowly releasing nanoparticles, while their mucoadhesive properties and acquisition of high viscosities at precorneal sites make them remarkable for delivering drugs to the eyes.

### 5.8 Nanomicelles

Nanomicelles are colloidal delivery systems with a hydrophobic core and a hydrophilic shell, ranging in size from 10 to 100 nm. Easy preparation, low toxicity, monodispersity, and high bioavailability are advantages of this system. Nanomicelles are water-soluble and have a high drug-coating capacity. They are capable of increasing the solubility of ocular drugs with limited water solubility in their hydrophobic core. This feature can lead to more efficient drug delivery in ocular tissues. Additionally, they efficiently enhance tissue permeation and minimize drug degradation [17,[143], [144], [145], [146], [147]].

Commercial suspensions (5 % Natamycin) exhibit quick precorneal clearance, limited corneal permeability, increased administration frequency, and corneal irritation due to undissolved particles. However, in a valuable study [148], natamycin-loaded nanomicelles (1 % Natcel) were formulated with transparency and stability properties. Compared with the 5 % suspension, 1 % Natcel showed superior corneal permeability. The formulation of 1 % Natcel provided a sustained release for up to 24 h. It was also biocompatible and had a longer mean residence time (MRT) in tears than the 5 % suspension. Consequently, 1 % Natcel may be a viable alternative treatment for fungal keratitis.

### 5.9 Nanoemulsion

Nanoemulsion is a colloidal system consisting of oil(s), water, surfactant(s), and co-surfactant(s) within the size range of 20–500 nm. The advantages of small-sized droplets include a higher active surface area, which results in more efficient ophthalmic drug delivery, improved ocular permeability, and higher bioavailability. Nanoemulsions possess the added advantage of reduced surface tension and superior drug dispersion over the cornea, facilitating effective integration with precorneal components. These attributes contribute to the prolonged drug contact duration on the corneal epithelium [[149], [150], [151], [152]].

Yeu et al. [153] evaluated the clinical effectiveness and safety of the propylene glycol/hydroxypropyl-guar (PG-HPG)-based nanoemulsion (Systane® Complete) for lubricating eye drops in patients with dry eye disease. In an IV phase study, adult dry eye cases were classified into three subgroups: aqueous deficient, evaporative, and mixed. A drop of PG-HPG was applied to each eye twice a day for 28 days. Briefly, the data demonstrated the efficacy of PG-HPG nanoemulsion eye drops for the treatment of dry eye disease.

### 5.10 Dendrimers

Dendrimers are artificial nanoconstructs with highly branched, star-shaped polymeric structures ranging from 5 to 20 nm in size. Considering the physicochemical properties of these carriers, such as molecular weight, size, surface charge, molecular geometry, and functional groups, they are considered a significant category in the concept of drug delivery. Dendrimers are suitable for delivering both hydrophobic and hydrophilic drugs due to their highly branched structure. Additionally, they enhance drug solubility and demonstrate high drug-loading capacity, along with sustained drug release characteristics. However, limitations such as blurred vision and potential loss of eyesight are associated with them [36,80,[154], [155], [156], [157], [158]].

In a prior study by Lin et al. [159], free dexamethasone (Free-Dex), dendrimer-dexamethasone (D-Dex), or saline were administered through a single subconjunctival injection to animals with induced autoimmune dacryoadenitis. A significant reduction in the number of pro-inflammatory genes (IL6, IL8, MMP9, TNFα) expression was observed in the D-Dex group compared to the Free-Dex and saline groups. This nanomedicine study for managing ophthalmic diseases (such as induced autoimmune dacryoadenitis) could be efficient for clinical applications.

### 5.11 Cyclodextrins

Cyclodextrins represent a group of cyclic oligosaccharides capable of forming inclusion complexes with various drugs. These complexes can adhere to the eye's surface, enhancing drug permeation and enabling sustained release. In addition, they can enhance drug solubility and mitigate drug irritation by controlling drug concentration. However, potential toxicity concerns at higher concentrations may limit their applicability [80,[160], [161], [162], [163], [164]].

Sohani et al. [165] developed an ophthalmic drop hydrogel utilizing curcumin nanoparticles encapsulated with β-cyclodextrin and hyaluronic acid to enhance corneal healing. Curcumin, which exhibits significant pharmacological qualities, including anti-cancer, anti-inflammatory, and antioxidant effects, is utilized in numerous medicinal formulations. The nanoparticles were assessed by in vitro investigations such as zeta potential, Fourier-transform infrared (FTIR), and scanning electron microscopy (SEM) analyses. Additionally, rabbit models of ulcerative keratitis were individually treated with topical drugs and assessed via various methods (such as fluorescein dye staining, corneal clarity score, and pathological assessments). In comparison with traditional treatment, the frequency of medication administration was reduced. Furthermore, improved tissue quality and a reduction in ulcerative keratitis (p < 0.05) were demonstrated. This study indicates the potential of novel ophthalmic topical medications for treating ulcerative keratitis.

**Fig. 3:**
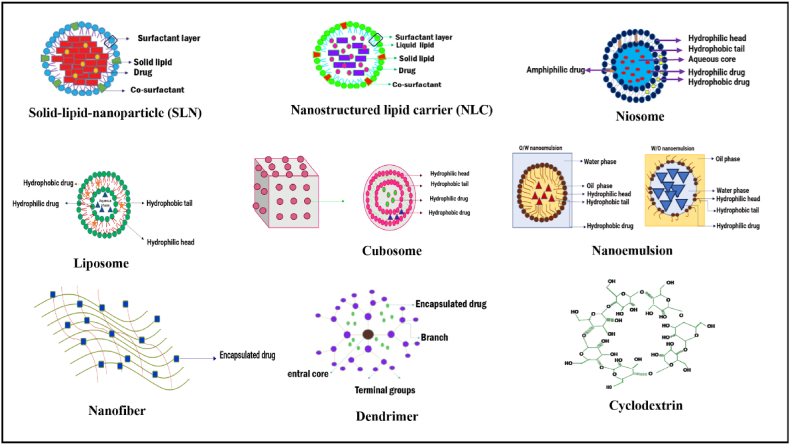
The chemical structures of drug carriers.

The chemical structure of nano-delivery systems is represented in (Fig. 3).

## 6 Engineered nanosystems for improved drug delivery

Various delivery systems have been used in numerous investigations to treat corneal disorders, frequently in modified or combined designs for drug loading in both laboratory and clinical situations [166]. These delivery systems can be manufactured using traditional technologies such as freeze drying, spray coating, micro milling, micromachining, photolithography, etching, deposition, mold replication, and assembly [167]. Alternatives include composite nanosystems that can simultaneously load and release medications or bioactive compounds over an extended time [168].

### 6.1 Engineered nanovesicles

Nanovesicles can be included in hydrogels (Exosome-loaded hydrogels, liposome-loaded hydrogels) for the benefit of protecting them against quick clearance, which improves their membrane integrity and mechanical toughness. Additionally, the physical, mechanical, and biological properties of hydrogels can be improved and customized by using these nanovesicle-infused hydrogels [168,169]. Piffoux et al. [170] fabricated personalized biosynthetic hybrid vectors by loading extracellular vesicles (EVs) into liposomes, thereby developing an advanced delivery system. Through this approach, fused exosomes were obtained using functionalized liposomes containing biological constituents such as membrane and soluble cargoes, alongside polyethylene glycol. These hybrid structures, enriched with biogenic molecules within their lipid bilayer and inner compartments, exhibited more therapeutic efficiency compared to either free drug or the drug-loaded liposome precursors. Another study conducted by Chang et al. [166]. administered the development of liposome-loaded hydrogels as a dual drug delivery system. Their formulation encapsulated antibiotics moxifloxacin (MFX) and dexamethasone (DEX) using nanostructured lipid carriers, which were integrated with a biodegradable material composed of collagen, gelatin, and alginate (CGA).

The utilization of the CGA-Lipo-MFX/DEX complex in an animal model yielded positive outcomes without observed side effects or toxicities. Moreover, this complex demonstrated the inhibition of pathogenic microorganism growth and enhancement of corneal wound healing. The evaluated results suggest that this anti-inflammatory formulation holds promise as a potential treatment for the ophthalmological disease.

Additionally, the integration of hybrid exosome-liposome particles into smart hydrogel systems, responsive to different stimuli such as chemical, biological, electrical, photo, thermo, or pH changes, presents a viable avenue for achieving more sustained and controlled drug delivery [168].

### 6.2 Metal-based nanosystems

Ceria-based nanosystems have been also developed to alleviate dry eye symptoms. Yang et al. [171] synthesized an engineered multifunctional nanoceria to deliver gabapentin in the presence of gelatin and glutaraldehyde. The structure exhibited antioxidative, antiangiogenic, anti-inflammatory, antiapoptotic, and biocompatible properties. Gelatin's abundant thiol groups enhanced cellular uptake by 2.3-fold and efficient mucin binding (10-fold), increasing ocular retention and therapeutic activity. A rabbit model of dry eye showed that the nanoformulation improved tear production. It also repaired the corneal epithelium defect, maintained corneal nerve density, and was more effective than free drugs. In summary, a potent and safe nanoformulation was proposed to reduce dry eye symptoms.

Nguyen et al. [172] investigated amination levels in chitosan coatings to enhance the penetration of ceria nanocapsules through ocular barriers to effective drug release. In addition to enhancing the carriers' burst release capabilities, high amination levels increased permeability through corneal cell layers by approximately 43-fold. Pilocarpine-loaded chitosan-coated ceria nanocapsules were used to treat an experimental rabbit model of acute glaucoma. Within 4 h, the treatment successfully reduced high intraocular pressures to normal levels, prevented retinal degeneration, opened the angle closure, and inhibited endothelium damage. This solution for the treatment of acute glaucoma and other urgent medical conditions may be available in the form of nano eye drops.

A nanomedicine approach was conducted to modify surface roughness-controlled ceria nanocages (SRCNs) and poly(l-histidine) surface coatings to activate various bioactive functions of intrinsically therapeutic nanocarriers, facilitate transport across the barriers of corneal epithelium, and release of dual drugs [acetylcholine chloride (ACh) and SB431542] at the injury site [173]. Briefly, surface modifications enhanced corneal penetration and enabled the smart release of drugs in damaged tissue. In vivo observations confirmed the efficiency of a single-dose topical nanoformulation, as shown by a reduction in corneal wound sites and approximately 93 % inhibition of abnormal blood vessel formation in a rat model of alkali burn (19-fold better than a commercial eye drop). Furthermore, corneal transparency was almost fully restored four days after administration. This nanoformulation was suggested to provide multifunctional metallic nanotherapeutics for tissue regeneration and ocular pharmacology.

In the management of ocular hypertension, an enormously common ocular condition, Y-27632 is an effective ophthalmic agent [174]. However, continuous administration of Y-27632 via a therapeutic carrier to damaged sites in the eye's inner segments remains a challenge. In a prior study, shell thickness influenced Y-27632 release patterns. This research showed that hollow mesoporous ceria nanoparticles (HMCNs) with thick (∼40 nm) and thin (∼10 nm) shells exhibited burst release properties (within 2 days) or had less drug-loading capacity. The longest drug release occurred in HMCNs with a shell thickness of approximately 20 nm, lasting 10 days. In a rabbit model of glaucoma treated with Y-27632-loaded HMCNs, about 86 % of compromised photoreceptor cells were restored. It was suggested that HMCN shell thickness is significant in the development of long-acting nano eye drops for glaucoma management. Nanobiomaterial structural engineering may play a significant role in pharmacologically managing intraocular diseases by developing long-lasting eye drops.

### 6.3 Metal-organic frameworks (MOFs)

Metal-organic frameworks (MOFs) are novel drug delivery systems comprising crystalline inorganic-organic hybrids. They offer potential encapsulating and delivering imaging agents and small-molecule drugs. However, challenges include potential linker toxicity, synthesis conditions, and pore size limitations. Huang et al. addressed these by developing nanoassembly carriers through coordinative interactions between imidazole of His6 and zinc ions, eliminating traditional ligands [115]. His6-metal assemblies (HmA) exhibited benefits like high loading capacity, pH-dependent release, low cytotoxicity, rapid endocytosis, and intracellular release. In treating anterior segment diseases, Wang et al. [65] utilized HmA in eye drops. For instance, they encapsulated dexamethasone sodium phosphate (Dexp) within nanosized particles (Dexp@HmA) to treat alkali burn inflammation. In vitro and in vivo results showed Dexp@HmA features like high encapsulation efficiency, high drug payload, pH-responsive drug release, and low cytotoxicity. Furthermore, its capability to control sustained drug release is associated with increased therapeutic efficiency in both in vitro and in vivo models. Notably, HmA emerges as a promising approach for addressing diverse anterior segment eye diseases.

### 6.4 Polymersomes

Polymersomes are nanocarriers to the load and release of hydrophilic and hydrophobic drugs. these nanostructures contain hydrophilic lumens which are encased with membranes of hydrophobic bilayer. Their low permeability, however, may make payload release difficult. To overcome this obstacle, redesigned polymersomes with improved applicability, such as stimuli-responsive versions, must be designed [175,176]. As an illustration, photoresponsive polymersomes with excellent efficiency have been used to accelerate corneal wound healing [176].

Different drug delivery systems for the treatment of corneal diseases are evaluated in (Table 3).

**Table 3:** Chemical drug delivery systems for healing corneal diseases.

Drug carriers	Drug	Role of Drug	Administration	Outcomes	In vitro/in vivo	Ref.
mPEG-b-PLGA NPs	TAC	A hydrophobic drug with low corneal penetrability that inhibits corneal allograft rejection after transplantation by reducing the IL-2, IL-17, and VEGF expression levels in the cornea and aqueous humor.	Eye drops and subconjunctival injection	- This study aimed to enhance transcorneal permeation and absorption of drugs. - This system had the potential to treat ocular diseases, by increasing the efficacy of hydrophobic drugs.	Rabbits and rats	[177]
SLNs	NAT	An agent against Fungal keratitis		- NAT-SLNs in a prolonged drug release rate improved corneal penetration and enhanced antifungal activity without cytotoxic effects on corneal tissues. - The designed system as a promising ocular delivery system for treating deep corneal keratitis could be considered.	Ex vivo	[178]
NLCs	Curcumin	It is a potent agent with antioxidant, anti-inflammatory, and anti-infectious effects. Curcumin has poor bioavailability due to its low aqueous solubility and stability which limits its clinical efficacy.		These results demonstrated curcumin permeation enhancement in excised corneas.	In vitro and ex vivo	[179]
Niosomes in ketorolac tromethamine gels	NAT	It is useful for healing fungal keratitis	Topical	To improve the corneal permeability and enhance the prolonged delivery of NAT is considered.	Rabbit model (keratitis)	[180]
Cubosomal NPs	Fluconazole	It is a bistriazole antifungal agent to treat ocular mycoses. However, it has poor permeation into ocular tissues and has a short half-life.	Topical	An enhanced antifungal activity of this drug for the treatment of fungal keratitis was observed.	Rat eyes (fungal keratitis)	[181]
Hyalugel-integrated liposomes	Fluconazole	It is a bistriazole antifungal agent for the treatment of ocular mycoses as an eye drop. However, it has poor permeation into ocular tissues and has a short half-life.	Topical	- An enhanced corneal permeability and increased antifungal activity were reported. - It would be a good candidate for ocular applications.	Ex vivo/in vivo (Rabbit eyes)	[182]
Electrospun nanofibers	CsA	An immunosuppressive drug to control the rejection of organ transplants and to treat inflammatory and various autoimmune conditions.	Suture	This carrier system was effective in suppressing corneal inflammation and corneal neovascularization.	Rabbit model (corneal alkali injury)	[138]
PLGA-PEG-PLGA thermosensitive hydrogel	MET- LFH	Inhibition factors for corneal neovascularization.	Subconjunctival injection	Co-polymers which showed great potential for ophthalmic anti-angiogenic therapy.	Mouse model (corneal alkali burn)	[183]
Nanomicelles based on PVCL-PVA-PEG	Curcumin	It is a potent agent with antioxidant, anti-inflammatory, and anti-infectious effects. Curcumin has poor bioavailability due to its low aqueous solubility and stability which limits its clinical efficacy.	Topical and/or intranasal	Nanomicelle curcumin was used for the treatment of corneal epithelial/nerve wound healing in diabetic keratopathy.	Mice (diabetic model)	[184]
Dendrimer	DEX	A synthetic corticosteroid, with anti-inflammatory effect to treat eye injuries.	Injectable gel	- This platform demonstrated the ability to improve the retention time of the drugs for the treatment of many inflammatory ocular surface disorders such as dry eye, auto-immune keratitis, and post-surgical complications.	Rat model (mild alkali burn)	[185]
HPβCD	TAC	To reduce inflammatory activity in different ocular diseases such as dry eye, uveitis, and corneal graft rejection is suggested.	Topical	A topical delivery system with a high potential for ophthalmic diseases was considered.	Rat eyes	[186]

**Abbriviations:** mPEG-b-PLGA NPs: methoxy poly (ethylene glycol-block-poly (D, L)-lactic-co-glycolic acid) nanoparticles, TAC: tacrolimus, IL2: interlucin 2, IL17:interlucin 17, VEGF: vascular endothelial growth factor, 2-HP-β-cyclodextrin- Polylactic-co-glycolic acid nanoparticles, TA: Triamcinolone Acetonide, SLNs: solid lipid nanoparticles, NAT: natamycin, NLCs: nanostructured lipid carriers, CsA: Cyclosporine A, PLGA-PEG-PLGA: poly(D,L-lactic-co-glycolic acid)-block-poly(ethylene glycol)-block-poly(D,L-lactic-co-glycolic acid), MET: Metformin, LFH: Levofloxacin Hydrochloride, PVCL-PVA-PEG: polyvinyl caprolactam-polyvinyl acetate-polyethylene glycol, DEX: Dexamethasone, HPβCD: Hydroxypropyl-β-cyclodextrin.

## 7 Drug-free systems

Various modification techniques have been applied to improve the pharmacokinetic properties of nanocarriers, aiming to enhance their efficiency in encapsulation, conjugation, transportation, and drug delivery [187]. However, limitations like inadequate effectiveness due to cellular barriers, multidrug resistance mechanisms, burst release, and potential organ toxicity, such as kidney damage, may be accompanied by drug-loaded carriers [[188], [189], [190]].

The intricate process of loading drugs often involves complex synthesis methods and the use of specific materials, increasing costs and diminishing the stability of drug delivery systems. Additionally, challenges such as batch-to-batch inconsistencies can hinder their clinical application [[191], [192], [193], [194]]. Despite these issues, many drug delivery systems still suffer from limited drug loading capacities, leading to unsatisfactory therapeutic outcomes [195]. Thus, the development of drug-free systems has emerged as a viable option for disease management, including inflammation and cancer therapy [187,196]. Luo et al. [197] employed a bioadhesive metallic nanotherapeutic system, utilizing gelatin-capped silver nanoparticles (G-Ag NPs), which exhibited dual antimicrobial and antiangiogenic properties for treating Staphylococcus aureus-induced keratitis. The in vitro and in vivo studies demonstrated a potent autoantigenicity efficiency of G-Ag NPs. Also, the system exhibited excellent corneal stromal residence, stability, antibacterial activity, biocompatibility, and significant bioactivity.

Several drug-free hydrogels are marketed for use as artificial tears or sealants. Various artificial tears have been used to treat dry eyes, such as Viscotears®, Liquivisc®, Clinitas Gel®, GelTears®, and Xailin Gel® [198,199]. Lin et al. [200] developed a long-lasting eye drop formulation for topical dry eye disease therapy using a drug-free carbonized nanogel (CNG) by pyrolysis of lysine hydrochloride (Lys). Lys-CNGs enhanced ocular bioavailability and precorneal retention. Additionally, in vitro and in vivo biocompatibility evaluations of Lys-CNGs demonstrated their safety as ocular drops. A single dose of Lys-CNGs (50 μg mL⁻^1^) in a rabbit dry eye model significantly reduced symptoms within 4 days. However, compared to cyclosporine A, multiple doses with 10 times higher content were required to achieve the same therapeutic effects (one dose every 12 h; 500 μg mL⁻^1^). Compared to commercial cyclosporine A eye drops, Lys-CNGs exhibited enhanced therapeutic efficacy due to reduced therapeutic dosages and prolonged dosing intervals. Lys-CNGs possess strong anti-inflammatory, free radical-scavenging, biocompatibility, antioxidant, and ocular bioadhesive properties, suggesting they could be used to develop long-lasting eye drops for treating dry eye disease.

In another study by Jiao et al. [201] an innovative antibacterial and antioxidant contact lens was fabricated using a polyacrylamide semi-interpenetrating network hydrogel comprising quaternized chitosan and tannic acid (PAM-QCS-TA). The synthesized hydrogels exhibited swelling resistance, high water content, light transmittance, and tunable mechanical properties. In antibacterial assays against *Staphylococcus aureus* and *Escherichia coli* (*E. coli*), great sterilization was demonstrated (particularly against *E. coli*). The presence of tannic acid in the structure conferred high antioxidant properties, protecting cells from reactive oxygen species cytotoxicity. Animal studies showed a reduced treatment duration for bacterial keratitis (within 3 days) and eye tissue preservation. Consequently, a contact lens with drug-free antibacterial and antioxidant properties and emergence of drug resistance prevention was developed. This efficient contact lens as a promising option for managing inflammatory diseases and ocular infections was suggested.

## 8 Designing drug delivery systems by emerging technologies

Administering dosage specifically at pathological sites can significantly enhance the efficiency of drug delivery systems [167,202]. Therefore, the utilization of advanced platforms such as microfluidic devices and 3D printing to optimize drug delivery processes with minimal side effects, achieving reproducible, on-demand, and tunable dosing at the required timing for patients while avoiding repetition, holds great promise [203]. 3D printing is one of the revolutionary formulation-preparation processes needed to enable the development of customized medicine. To create 3D objects, printing materials are deposited layer by layer using a computer-aided design method, which may be efficient in developing personalized medications [204].

The development of recent microfluidic lab-on-a-chip technology has provided unprecedented opportunities for precise drug delivery through the integration, implantation, localization, automation, and meticulous control of various microdevice parameters. An illustrative study showed [205], the sub-tenon microfluidic system (SMS) in regulating the pharmacokinetics of plasma and ocular dexamethasone (DEX) for delivering dexamethasone sodium phosphate (DEXP) in rabbits with experimental autoimmune uveitis (EAU). Employing a miniature pump connected to a tube implanted within the sub-tenon's sac of EAU rabbits facilitated the sub-tenon microfluidic system (SMS) to exhibit sustained DEX release and a notable drug loading capacity. Comparative to the conventional approach of combined medication (subconjunctival injection combined with intravenous injection) in EAU rabbits, the SMS showcased an enhanced distribution of DEX throughout ocular tissues. Notably, this method limited drug entry into the bloodstream, thereby minimizing systemic adverse effects. With its potential to potentially replace or eliminate the need for systemic medications in managing acute ocular inflammatory conditions, the SMS presents itself as a viable choice for ocular clinical trials.

Moreover, the utilization of 3D printing for dosage form manufacturing has garnered substantial interest in the realm of pharmaceutical technology. The field of pharmaceutical technology has seen significant interest in the utilization of 3D printing for manufacturing dosage forms [206]. The mechanism of this technology is the fabrication of 3D objects from digital models by deposing layer-by-layer printed materials.

Sophisticated 3D printing methodologies hold substantial promise in the fabrication of intricate dosage forms with diverse profiles of drug release, thereby facilitating the tailoring of pharmaceutical formulations [207]. The expedited production of personalized pharmaceutical formulations, encompassing patient-specific variables such as age, weight, organ functionality, and disease severity, is a notable feature of 3D printing technology. The execution of this process is facilitated through the application of computer-aided design (CAD) files [202,208]. Stereolithography, selective laser sintering, inkjet-based 3DP, fused deposition modeling, and pressure-assisted microsyringe are among the 3D printing methods utilized in the pharmaceutical domain [202,207]. These techniques have been explored for enhancing drug delivery efficiency and achieving sustained release profiles.

In a prior study [209], 3D printing technology has been employed in the pharmaceutical realm. In this investigation, innovative ophthalmic patches for controlled drug release are developed using a semi-solid material extrusion-type 3D printer. Printer inks based on hydrogel consisting of hypromellose, sugar alcohols (mannitol, xylitol), and drugs, were utilized to construct patch-shaped structures through 3D printing, followed by a freeze-drying process. 3D-printed structures were characterized, based on physical properties, surface structure, water uptake, antimicrobial activity, and drug release profile of lyophilized patches. Afterward, based on patient-tailored dosages, different dosages, and patterns were obtained containing multiple drugs for clinical applications.

Digital light processing (DLP) 3D printing was used to fabricate dexamethasone-loaded punctal plugs previously [210]. A semi-interpenetrating network (semi-IPN) was established using polyethylene glycol diacrylate (PEGDA) and polyethylene glycol 400 (PEG 400). An evaluation of drug-loaded punctal plugs was conducted for cytocompatibility, drug release profile, potential drug-photopolymer interactions (FTIR), and physical properties (XRD and DSC). According to the results, 20 % w/w PEG 400 and 80 % w/w PEGDA punctal plugs released dexamethasone continuously for up to seven days, whereas 100 % PEGDA punctal plugs released it continuously for over 21 days. Collectively, DLP 3D printing was offered as a viable manufacturing platform to fabricate customized punctal plugs with sustained medication release for ocular administration.

In another study, 3D-printed inserts containing liposomal moxifloxacin (SL: MOX) to improve drug penetration and ocular contact time were fabricated [211]. Through dynamic light scattering, moxifloxacin liposomes were measured at 150 nm. This study showed that 80 % of moxifloxacin could be encapsulated within lecithin liposomes (SL: MOX). An ex vivo investigation was conducted to assess the antibacterial susceptibility of MOX, SL: MOX, and a commercial product. The ocular inserts were then designed and optimized using sodium hyaluronate (NaH) through an extrusion 3D printing method. Liposomes improved the solubility of moxifloxacin, and the 3D-printed insert released the medication noticeably slower than the control. A 3D printer produced ocular inserts (moxifloxacin and lecithin liposomes) with stability and superior content uniformity by incorporating liposomal moxifloxacin. Based on 3D printing, customized eye medications can be developed specifically for each patient with eye diseases.

Besides, a dissolving ocular microneedle patch using an optimized stereolithography (SLA) 3D printing method was produced [212]. The microneedle architecture and printing precision were analyzed in terms of layer thickness, aspect ratio, mold geometry, length, and printing orientation. For a variety of fundamental shapes, including pyramidal, conical, and triangular pyramidal, the effects of the printing angle on needle fidelity were also evaluated. In vitro mechanical and penetration tests were conducted on polymeric polyvinyl pyrrolidone and polyvinyl alcohol (PVP/PVA) microneedle patches, fabricated from reverse molds in a variety of heights and shapes, and aspect ratios. An ex vivo penetration test was further performed on excised ocular tissues (cornea and sclera). Through optimization, the parameters required to produce microneedles with the highest dimensional fidelity and sharpest tips were determined. According to ex vivo studies, these optimized systems to penetrate ocular tissue with minimal pressure and easy self-administration for patients have potential.

In general, designing structures using biomaterials and tissue engineering has been studied extensively. Moreover, the fabrication of mimicked microenvironments has garnered significant interest in tissue regeneration [[213], [214], [215]]. In drug delivery system development, a polymer biomaterial's interaction with a drug enhances therapeutic efficacy, improves drug stability, and reduces local immune responses [92].

**Fig. 4:**
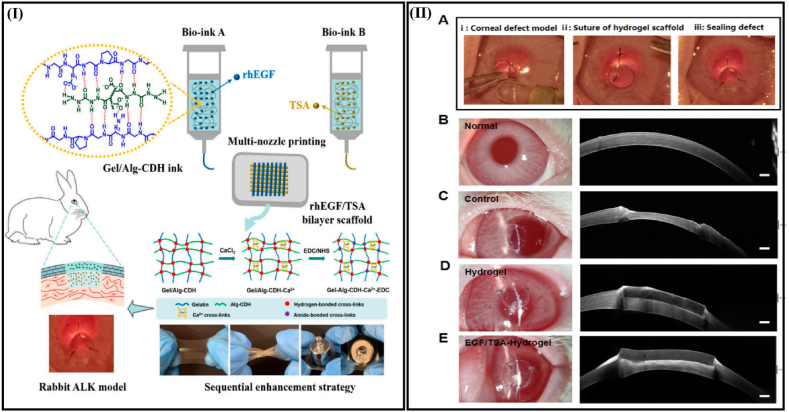
(I) Schematic representation of the Gel/Alg-CDH inks used to 3D-printed hydrogel bilayer scaffolds loaded with rhEGF/TSA for rabbits with corneal ALK models. (II) The evaluation of 3D-printed hydrogel scaffolds containing rhEGF/TSA in rabbit ALK model. (A) The ALK procedure involves the removal of the corneal epithelium and partial of the corneal stroma, followed by hydrogel scaffold implantation and suturing. (B–E) The slit lamp images (left) and the AS-OCT images (right) show the normal cornea, the defected cornea (control group), the implantation of hydrogel group, and the implantation of rhEGF/TSA bilayer hydrogel group, respectively.

3D (bio)printing, by fabricating mimicked structures similar to native tissues, is also highly applicable for regenerative medicine applications. For instance, in a prior study by Nie et al. [216] 3D printing was used to fabricate mimicked scaffolds of natural polymer biodegradable hydrogel to promote corneal tissue regeneration. A polymer hydrogel ink was made from concentrated aqueous solutions of gelatin and carbohydrazide-modified alginate (Gel/Alg-CDH). Extrusion printing with hydrogel Gel/Alg-CDH exhibited appropriate viscosity thickening, shear thinning, and improved mechanical properties. Moreover, superior transparency, swelling stability in physiological conditions, suturability, and rapid degradation by enzymes were demonstrated in Gel-Alg-CDH-Ca^2+^-carbodiimide hydrochloride (EDC) hydrogels. With multi-nozzle printing, the scaffold for a biomimetic bilayer hydrogel can be personalized to carry growth factors and drugs. Through rabbit corneal keratoplasty, this scaffold induced corneal epithelium regeneration and inhibited corneal scarring. Fig. 4 (I) shows the application of extrusion printing for development of bilayer scaffolds using Gel/Alg-CDH inks. A hydrogel bilayer scaffold loaded with recombinant human epidermal growth factor layer (rhEGF)/Trichostatin A (TSA) in rabbit corneal models is also presented in this figure.

Fig. 4 (II) shows the efficiency of 3D-printed bilayer scaffolds containing rhEGF/TSA to repair corneal defects in the rabbit model with anterior lamellar keratoplasty (ALK) [216].

ALK: anterior lamellar keratoplasty; rhEGF: recombinant human epidermal growth factor layer; TSA: Trichostatin A (Reprinted with some modifications from Ref. [216]).

By combining artificial intelligence and advanced technologies (such as 3D printing), the recent transformation of the medical industry is expected to facilitate the design and development of customized medications. Hu et al. [217]. presented the design and manufacturing of personalized capsules incorporating AI and 3D printing technology. After discretizing and encoding the capsule geometry, a traditional drug dissolution model was used to simulate the capsule's disintegration.

A genetic algorithm was then employed to investigate the geometric structure space of the capsules and produce a sophisticated multi-layer configuration that meets the specified drug release characteristics. The AI-designed capsule was precisely printed using fused deposition modeling technology, employing two model medications, isoniazid and acetaminophen. By altering the capsule's form, it was possible to design an accurate and independent release curve for the drug. An in vitro comparison between the printed capsules' release curve and the target curve confirmed the method's reliability. The method is expected to provide individualized dosage forms for various medications.

## 9 Conclusions

The topical prescription of conventional systemic medications is often challenged by rapid excretion and degradation processes, leading to inadequate drug accumulation at desired anatomical sites. Innovative drug delivery systems, encompassing nanoparticles, liposomes, niosomes, and dendrimers, exhibit remarkable potential in addressing these limitations by facilitating controlled and sustained drug release, thereby minimizing untoward effects and optimizing therapeutic outcomes.

Moreover, the pursuit of tailored drug delivery systems with specific release kinetics for localized medication administration, achieved through strategies such as integration, implantation, and localization, holds substantial appeal. The emergence of such personalized platforms offers an intriguing avenue for exploration, allowing for enhanced precision and effectiveness in therapeutic interventions. For instance, 3D printing technologies enable customization of drug delivery systems in terms of size, shape, and release profile, catering to specific patient populations such as pediatrics, pregnant women, and geriatrics based on their genetic and genomic attributes. These versatile structures, including tablets, scaffolds, implants, microneedles, capsules, films, hydrogels, mouthguards, tubes, stents, and rings, hold promise for applications in personalized medicine.

## CRediT authorship contribution statement

**Mahsa Fallah Tafti:** Writing – original draft, Investigation, Data curation, Conceptualization. **Zeinab Fayyaz:** Writing – review & editing, Investigation. **Hossein Aghamollaei:** Writing – review & editing, Validation, Project administration, Conceptualization. **Khosrow Jadidi:** Writing – review & editing, Validation, Project administration, Conceptualization. **Shahab Faghihi:** Writing – review & editing, Supervision, Project administration.

## Data availability statement

Data will be made available on request.

## Ethical statement

This work does not need an ethical statement.

## Funding

No funding was received for this study.

## Declaration of competing interest

The authors declare that they have no known competing financial interests or personal relationships that could have appeared to influence the work reported in this paper.
